# Social environment modulates investment in sex trait versus lifespan: red deer produce bigger antlers when facing more rivalry

**DOI:** 10.1038/s41598-020-65578-w

**Published:** 2020-06-08

**Authors:** Juan Carranza, Javier Pérez-Barbería, Concha Mateos, Susana Alarcos, Jerónimo Torres-Porras, Javier Pérez-González, Cristina B. Sánchez-Prieto, Juliana Valencia, Leticia Castillo, Eva de la Peña, Isabel Barja, José M. Seoane, Manuel M. Reglero, Antonio Flores, Alberto Membrillo

**Affiliations:** 1 Wildlife Research Unit (UIRCP), Universidad de Córdoba, 14071 Córdoba, Spain; 2 Biology and Ethology Unit, Universidad de Extremadura, 10071 Cáceres, Spain; 3 Department of Social and Experimental Sciences Teaching, Faculty of Educational Sciences, Universidad de Córdoba, 14071 Córdoba, Spain; 4 Didáctica de las Ciencias Experimentales, Facultad de Ciencias de la Educación, Universidad de Málaga, Málaga, Spain; 5 Department of Biology, Zoology Unit, Universidad Autónoma (UAM), 28049 Madrid, Spain; 6 Gestión Cinegética Integral S.L. and Lagunes Selección Genética S.L., Madrid, Spain; 7 Game and Livestock Resources Unit, University of Castilla-La Mancha, IDR, IREC, Albacete, 02071 Spain; 8 Biodiversity and Global Change Research Centre (CIBC-UAM), Universidad Autónoma (UAM), 28049 Madrid, Spain

**Keywords:** Ecology, Evolution, Zoology

## Abstract

Theory predicts that the plastic expression of sex-traits should be modulated not only by their production costs but also by the benefits derived from the presence of rivals and mates, yet there is a paucity of evidence for an adaptive response of sex-trait expression to social environment. We studied antler size, a costly and plastic sex trait, and tooth wear, a trait related to food intake and longevity, in over 4,000 male Iberian red deer *(Cervus elaphus hispanicus)* from 56 wild populations characterized by two contrasting management practices that affect male age structure and adult sex-ratio. As a consequence, these populations exhibit high and low levels of male-male competition for mating opportunities. We hypothesized that males under conditions of low intra-sexual competition would develop smaller antlers, after controlling for body size and age, than males under conditions of high intra-sexual competition, thus reducing energy demands (i.e. reducing intake and food comminution), and as a consequence, leading to less tooth wear and a concomitant longer potential lifespan. Our results supported these predictions. To reject possible uncontrolled factors that may have occurred in the wild populations, we carried out an experimental design on red deer in captivity, placing males in separate plots with females or with rival males during the period of antler growth. Males living with rivals grew larger antlers than males living in a female environment, which corroborates the results found in the wild populations. As far as we know, these results show, for the first time, the modulation of a sexual trait and its costs on longevity conditional upon the level of intra-sexual competition.

## Introduction

The expression of many sex-traits is plastic and responds to individual- and population-specific reaction norms^1–6^. There is evidence for many species that environmental factors (e.g. resource availability) affect body condition and the expression of sex-traits, such as signals and weapons^1,7–11^. Condition-dependence theory relies on the relative value of energy costs, because costs of producing traits are expected to be lower for individuals in good condition as compared to those in poor condition^12–15^. But also, in a sexual selection context, rivals and potential mates in the social environment strongly influence the benefits associated with trait expression, so that trait investment should respond in a trade-off fashion depending on the costs but also on the benefits of trait development. One example of this in the inter-sexual selection context is that male zebra finches invest more in coloring their beaks when there are females to receive the signal^16^. For intra-sexual competition, the challenge hypothesis^2^ applied this idea to testosterone production relative to the probability of winning contests, which may be related to sex-trait expression^17–20^. However, trait development may depend on other costs and benefits not necessarily linked to testosterone production^21^. Thus, testing how individuals modulate sex-trait expression to maximize fitness gains under a particular social environment relative to the associated costs in maintenance and survival remains a challenge.

Deer antlers are plastic traits that are grown and discarded every year, represent a considerable percentage of an animal’s skeletal mass, and their production is highly energy-demanding^22^. There is evidence that indicates that deer with more worn teeth relative to age grow larger antlers^23^ and have a larger skeletal body size^24,25^, which suggests that tooth wear is a proxy of food intake investment. But at the same time, because teeth in deer cannot be replaced or repaired during the animal’s life, tooth wear becomes a proxy of animal lifespan^26,23–28^. Under this scenario, a social environment that favours male-male competition for mating partners is expected to lead to deer producing larger antlers, but at a cost of shortening life expectancy, because tooth duration is compromised by tooth wear.

According to theory, we should expect red deer antlers to be smaller, either when their bearer is in poor condition (condition-dependence), or when the fitness return related to antler size increase is low due to the presence of few male rivals competing for potential mates. Though there is some evidence for the positive relationship between body size or condition and antler size in support for the condition-dependence hypothesis in red deer^8^, no study has assessed the role of social environment (intra-sexual competition at population level).

Here we study antler size in relation to the level of male-male competition in red deer populations in Southern Spain, controlling for body size and age, and the effect on tooth wear, a trait related to lifespan. We used populations that presented variation in the number of male competitors relative to potential mates, which was reflected in actual population differences in intra-sexual competition^29,30^. In addition, we undertook an experimental design, in which males were controlled for age, body size and faecal hormone metabolites (testosterone and cortisol, see Methods), and were exposed to two social environments during the period of antler growth: no male-male competition, that is, one male living in a female group; and male-male competition, consisting of single-sex male group (we refer to HC for social environments with high male-male competition and LC for low competition ones, either in natural or captive deer experiment, see Methods). We hypothesised that males under conditions of low intra-sexual competition would develop smaller antlers, and hence experience lower food energy costs that would be reflected in less tooth wear, after controlling for body size and age, in comparison to males under higher intra-sexual competition.

## Results

### Natural experiment

#### Age structure in HC and LC populations

**Figure 1:**
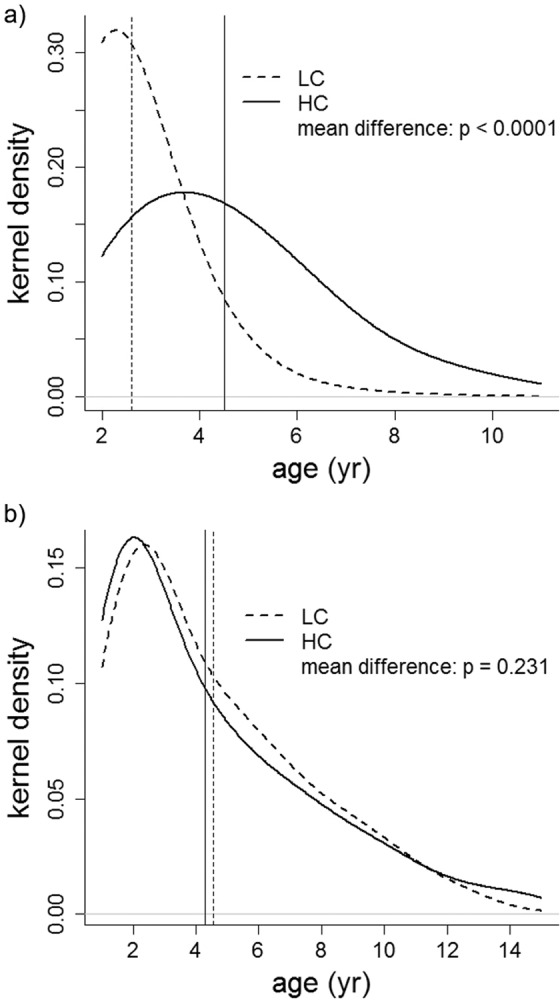
Kernel density of the age structure of shot red deer males (**a**) and females (**b**) from HC and LC populations. Vertical lines are the predicted mean age calculated using mixed linear models (see Methods). Graphics were carried out using the generic function plot in R^96^.

Male mean age was greater in HC than in LC populations. In HC the mean age of culled males was 4.51 yr (range = 2–11 yr, 1^st^ – 3^rd^ quartiles = 3–6, n = 2024), against 2.61 yr in LC (range = 2–9 yr, 1^st^ – 3^rd^ quartiles = 2–3, n = 2177; F_1, 40.67_ = 78.06, p < 0.0001, Fig. 1a). The proportion of males older than 2 yr, estimated from direct counts, corroborated that the age structure of males in HC populations were biased to older age in comparison with LC populations (males older than 2 yr in HC = 50.7% vs. LC = 16.9%, F_1, 27.5_ = 30.85, p < 0.0001).

There were no significant differences in the age structure of culled hinds between HC and LC. Their age ranged between 1 and 15 years (1^st^ – 3^rd^ quartiles = 2–6, predicted mean for HC = 4.29, n = 1286; predicted mean for LC = 4.55, n = 1209; F_1, 22.58_ = 1.52, p = 0.231, Fig. 1b).

Habitat assessment of areas occupied by HC and LC populations did not reveal any significant difference in habitat quality (Habitat quality index: HC = 258.2; LC = 273.6; F_1, 13.97_ = 0.94, p = 0.347).

#### Antler size and body weight in HC and LC populations

**Figure 2:**
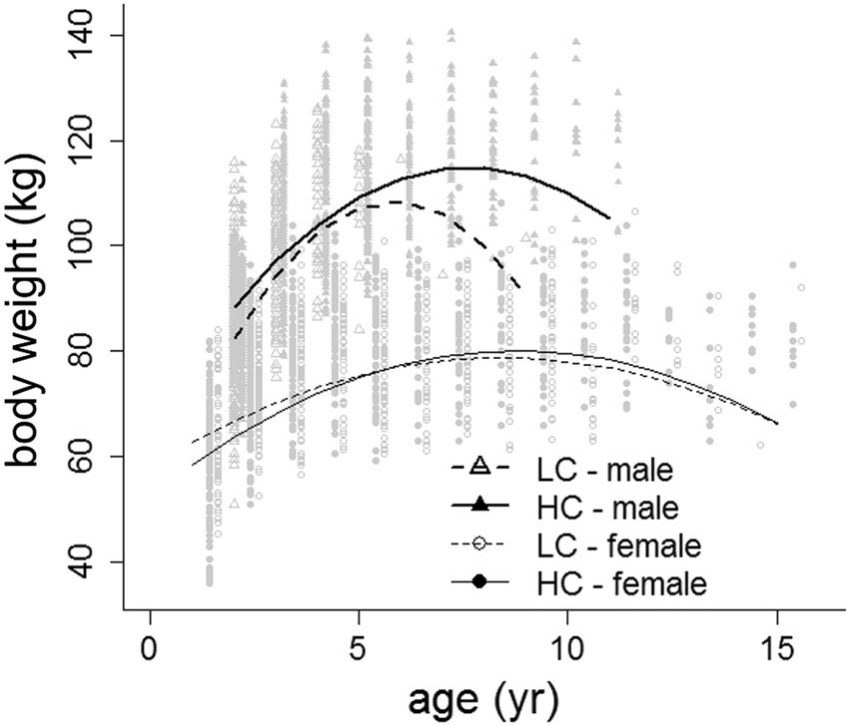
Predictions from model in Table 1 of body weight against age in males and females from populations with two levels of male-male competition for mating opportunities (high competition: HC; low competition: LC). Graphic was generated using the generic function plot in R^96^.

Males differed in body weight (BW) between HC and LC populations. The predicted maximum body weight in HC populations was 114.8 kg against 108.3 kg in LC populations, while in females the predicted maximum body weight in both types of populations was more similar (81.1 kg and 78.7 kg, respectively) (Table 1, Fig. 2). The rates of weight change with age were faster in males than in females in HC and LC populations, and within males, the body weight of LC populations decreased faster once body weight peaked. Males of LC populations reached their predicted prime body weight at 6 years old in comparison with 8 yr of age in HC populations. Females’ body weight peaked at 8 yr of age in LC populations and at 9 yr in HC populations, with very little variation across age between populations (Fig. 2). The model explained 64% of the variance by the fixed effects (*R*^2^_LMM(*m*)_, Table 1) and 77% (*R*^2^_LMM(*c*)_, Table 1) by the combined fixed and random effects. The standard deviation of the random effects indicates that there was more variation in body weight attributed to differences between populations than that due to variation between hunting seasons.

**Table 1:** Coefficients of the linear mixed-effects model on body weight (kg) for males and females from populations with two levels of male sexual competition for mates (i.e. social environment: high competition: HC; low competition: LC; reference level HC), controlling for population, hunting season and culling date (DOY: day of year, see Methods).

Random effects					
Groups	variance	std.dev.	Chi.sq	Chi.df	p
populations (intercept)	29.07	5.391	221	1	<0.0001
hunting season (intercept)	19.12	4.373	197	1	<0.0001
Residual	78.36	8.852			
N = 2781					
**Fixed effects**					
	**Estimate**	**std error**	**df**	**t-value**	**p**
intercept	64.38235	2.21343	62	29.087	p < 0.001
sine DOY	−1.49019	0.91814	2752.4	−1.623	0.10469
cosine DOY	−6.23236	0.36383	2755.7	−17.13	p < 0.001
age	4.88043	0.4044	2723.3	12.068	p < 0.001
age^2^	−0.28919	0.03314	2723.1	−8.726	p < 0.001
sex (male)	−10.0703	3.08023	2723.3	−3.269	0.00109
social environment	−5.56371	2.21441	73.1	−2.513	0.01419
age x sex (male)	15.82402	1.83535	2717.6	8.622	p < 0.001
age^2^ x sex (male)	−1.48781	0.24615	2715.5	−6.044	p < 0.001
age x social environment	1.3856	0.51556	2725.1	2.688	0.00724
age^2^ x social environment	−0.06746	0.04096	2724.1	−1.647	0.09972
sex (male) X social environment	23.71277	3.58064	2736.4	6.622	p < 0.001
age x sex (male) x social environment	−9.2768	1.97612	2719.6	−4.694	p < 0.001
age^2^ x sex (male) x social environment	1.00212	0.25418	2715.7	3.943	p < 0.001
R^2^_LMM(*m*)_	0.635747				
R^2^_LMM(*c*)_	0.774451

**Figure 3:**
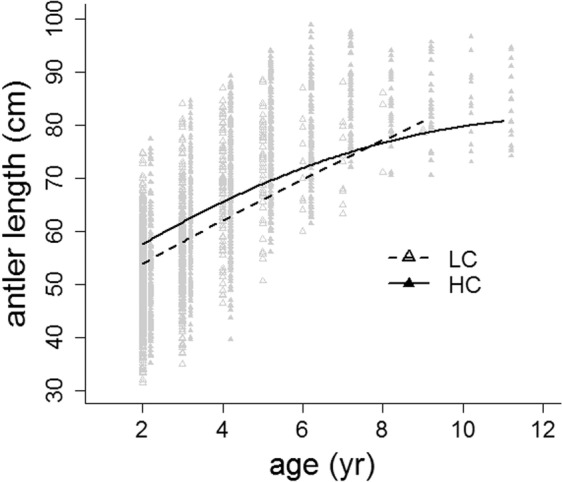
Predictions from model in Table 2 of antler beam length in males from populations with two levels of male-male sexual competition for mating opportunities (high competition: HC; low competition: LC). Graphic was generated using the generic function plot in R^96^.

For males of the same age and body size (as indicated by mandible length), those of HC populations had significantly greater antler length, on average 3.6 cm, than those of LC populations (Table 2; Fig. 3). Variation in antler length was greater between populations than between hunting seasons, as indicated by the standard deviation of the random effects (Table 2). Random and fixed effects together explained 80% of the variation in antler length.

**Table 2:** Coefficients of the linear mixed-effects model on antler beam length (cm) against age (yr) controlling for mandible length (ML, a proxy of body size, see Methods) in red deer males from populations with two levels of male sexual competition for mates in social environment (high competition: HC; low competition: LC; reference level HC).

Random effects					
Groups	Variance	std dev	Chi.sq	Chi.df	p
populations (intercept)	28.864	5.373	804	1	<0.0001
hunting season (intercept)	5.964	2.442	159	1	<0.0001
Residual	44.341	6.659			
N = 2718					
**Fixed effects**					
	**Estimate**	**std error**	**t-value**	**p**	
intercept	3.00926	30.26335	0.099	0.9208	
ML	−0.90172	2.27169	−0.397	0.6914	
ML^2^	0.08045	0.04277	1.881	0.0601	
age	8.63061	0.31874	27.077	<0.0001	
age^2^	−0.51762	0.02867	−18.053	<0.0002	
social environment	3.62958	1.76272	2.059	0.0458	
R^2^_LMM(*m*)_	0.640				
R^2^_LMM(*c*)_	0.798

**Figure 4:**
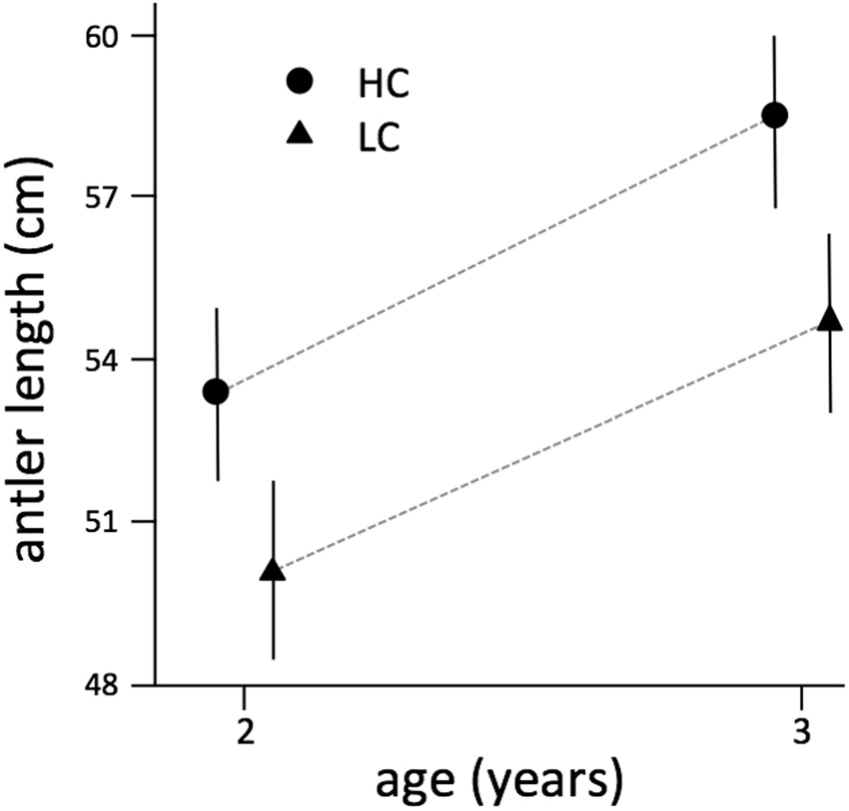
Predicted means of antler length (cm, ± se) of 2 and 3 year-old males in estates with high (HC) and low (LC) male-male competition for mating opportunities. Graphic was generated using the generic function plot in R^96^.

Trophy-size biased selection of two-year-old males was not stronger in LC compared with HC, since an analysis of variance of type III on the terms of the mixed linear model clearly indicated that the interaction between population type and age was not significant (F_1, 1510.8_ = 0.003, p = 0.957). Mandible length was not included in the final model as it was not significant, but the output of a full model that included mandible length and its interactions with age and HC/LC population type was consistent with this result. The predicted antler beam length for males of 2 and 3 years of age in HC and LC populations using model in Table 2 are shown in Fig. 4.

**Figure 5:**
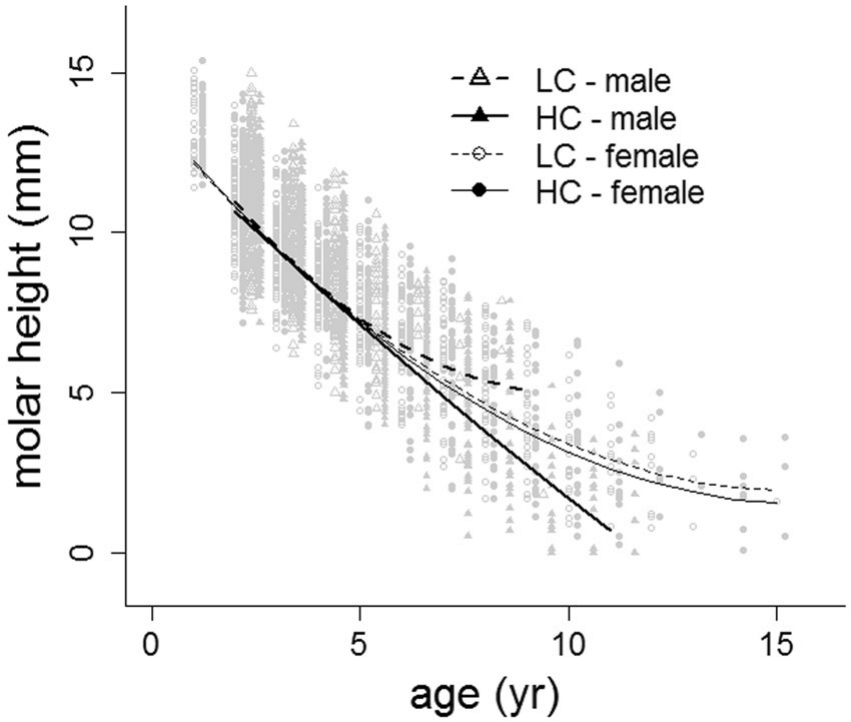
Predictions from model in Table 3 of molar height (a proxy of tooth wear) against age for males and females from populations with two levels of male-male competition for mating opportunities (high competition: HC; low competition: LC). (reference shot day was fixed at 26^th^ September). Graphic was generated using the generic function plot in R^96^.

Molar height, a measure inversely related to tooth wear, decreased with age in both sexes of HC and LC populations, and in general, the rate of tooth wear decreased as age increased (Table 3, Fig. 5). In both types of population, females had a very similar rate of tooth wear, but in males the pattern of tooth wear across age differed significantly between HC and LC populations (Table 3, Fig. 5). That is, in LC populations males’ rate of tooth wear decreased after 5 years of age, while the rate of tooth wear in males of HC populations was constant across age (Fig. 5), and males of HC populations had the lowest values of molar height among sexes and HC and LC populations, after controlling for age. The partitioning of the variance in tooth wear within the random effects, as indicated by the standard deviation of the random effects (Table 3), indicated that populations explained more variation than hunting seasons. The model explained a significant contribution of the variance of the data, 75% by the fixed effects (*R*^2^_LMM(*m*)_, Table 3), and 79% (*R*^2^_LMM(*c*)_, Table 3) by the combined fixed and random effects.

**Table 3:** Coefficients of the linear mixed-effects model on molar height (mm) (a proxy of tooth wear, see Methods) against age in red deer males and females from populations with two levels of male sexual competition for mates in social environment (high competition: HC; low competition: LC; reference level HC).

Random effects					
Groups	Variance	std dev	Chi.sq	Chi.df	p
populations (intercept)	0.2168	0.4656	135	1	<0.0001
hunting season (intercept)	0.1502	0.3876	185	1	<0.0001
Residual	1.6652	1.2904			
N, females = 1276, males = 2074					
**Fixed effects**					
	**Estimate**	**std error**	**t-value**	**p**	
intercept	13.620	0.2680	50.807	<0.0001	
age	−1.512	0.0720	−21.004	<0.0001	
age^2^	0.049	0.0059	8.283	<0.0001	
social environment	0.116	0.2966	0.392	0.696	
sex (male)	0.765	0.3368	2.273	0.023	
age x sex (male)	−0.375	0.1893	−1.979	0.048	
age^2^ x sex (male)	0.046	0.0241	1.896	0.058	
age x social environment	−0.039	0.0940	−0.420	0.674	
age^2^ x social environment	0.000	0.0074	0.048	0.961	
sex x social environment	−1.303	0.4375	−2.979	0.003	
age x soc env x sex (male)	0.648	0.2180	2.972	0.003	
age^2^ x soc env x sex (male)	−0.082	0.02587	−3.169	0.002	
R^2^_LMM(*m*)_	0.745				
R^2^_LMM(*c*)_	0.792

**Figure 6:**
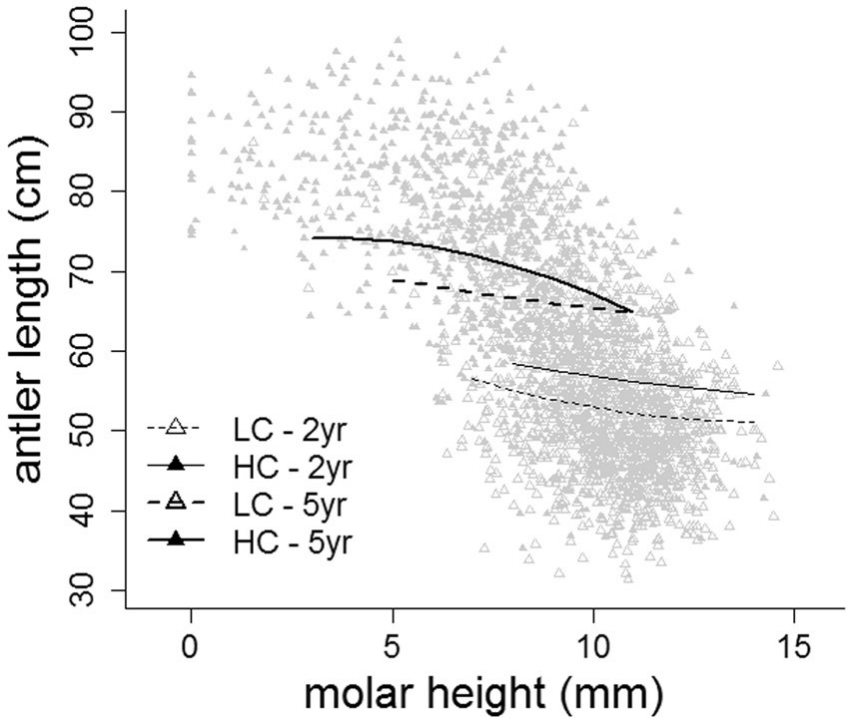
Predictions from model in Table 4 of antler beam length against molar height (a proxy of tooth wear) from populations with two levels of male-male competition for mating opportunities (high competition: HC; low competition: LC) at two arbitrary ages of 2 and 5 years old (mandible length, a proxy of body size, was fixed at its mean value). Graphic was generated using the generic function plot in R^96^.

Antler length was negatively related to molar height after controlling for the effects of age and mandible length (Table 4, Fig. 6). That is, for animals of the same age and similar body size, those with a greater tooth wear had larger antlers, and this effect was stronger in males of HC than in LC populations, especially for older animals (Table 4, Fig. 6). The contribution of the random effect population explained more than twice the amount of variance attributable to hunting season, as indicated by the random effects standard deviation (Table 4). The model explained 70% of the variance by the fixed effects (*R*^2^_LMM(*m*)_, Table 4) and 83% (*R*^2^_LMM(*c*)_, Table 4) by the combined fixed and random effects.

**Table 4:** Coefficients of the linear mixed-effects model on antler beam length (in cm) against molar height (MH) controlling for mandible length (ML, a proxy of body size, see Methods) and age in red deer males from populations with two levels of male sexual competition for mates in social environment (high competition: HC; low competition: LC; reference level HC).

Random effects					
Groups	variance	std dev	Chi.sq	Chi.df	p
populations (intercept)	25.56	5.056	741.,6	1	<0.0001
hunting seasons (intercept)	3.778	1.944	93.9	1	<0.0001
Residual	38.039	6.168			
N = 2504					
**Fixed effects**					
	**Estimate**	**std error**	**t-value**	**p**	
intercept	−85.732	47.6736	−1.798	0.072	
ML	8.653	3.1084	2.784	0.005	
ML^2^	−0.090	0.0581	−1.552	0.121	
age	−6.377	9.4228	−0.677	0.499	
age^2^	0.951	0.8403	1.132	0.258	
social environment	−16.647	25.1117	−0.663	0.507	
MH	−9.937	4.3773	−2.270	0.023	
MH^2^	0.658	0.2272	2.894	0.004	
age x social environment	8.371	9.8708	0.848	0.396	
age^2^ x social environment	−1.030	0.8603	−1.197	0.231	
age x MH	4.597	2.0322	2.262	0.024	
age^2^ x MH	−0.559	0.1982	−2.822	0.005	
age x MH^2^	−0.375	0.1183	−3.173	0.002	
age^2^ x MH^2^	0.049	0.0139	3.561	0.000	
soc env x MH	5.254	4.9468	1.062	0.288	
soc env x MH^2^	−0.345	0.2573	−1.342	0.180	
age x soc env x MH	−2.768	2.1273	−1.301	0.193	
age^2^ x soc env x MH	0.420	0.2031	2.068	0.039	
age x soc env x MH^2^	0.213	0.1247	1.711	0.087	
age^2^ x soc env x MH^2^	−0.036	0.0143	−2.483	0.013	
R^2^_LMM(*m*)_	0.695				
R^2^_LMM(*c*)_	0.828

## Captive deer experiment

**Figure 7:**
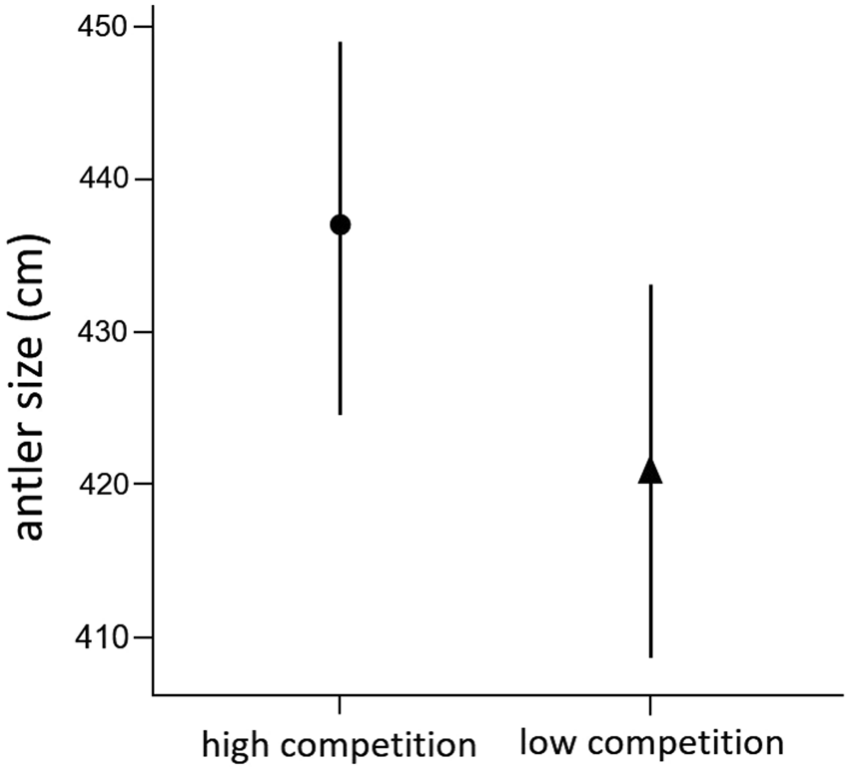
Predictions and standard errors on antler size of red deer males exposed to social environments of high and low male-male competition using Model A in Table 5. Graphic was generated using ggplot2^99^.

As was hypothesised, the social environment of low male-male competition depressed antler growth after controlling for herbaceous quality (NDVI index, see Methods), previous antler and body weight (Model A, estimate = −16.27, p = 0.035, Table 5). Out of these controlling terms only antler size of the previous year was positively associated with antler size (estimate = 0.95, p < 0.001, Table 5). The variance explained by the random effect year was not significant. The model explained a significant amount of the variance of the system (*R*^2^_LMM(*c*)_ = 0.88, *R*^2^_LMM(*m*)_ = 0.74). The prediction of the effect of social environment on antler size when all other fixed effects were fixed to their mean values was 437 cm (se = 12.3) in the high intra-sexual competition environment, and 421 cm (se = 12.4) in the low competition environment (Fig. 7).

**Table 5:** Coefficients of a linear mixed model (Model A) on red deer antler size (cm) against NDVI, previous antler (size of the antler grown in the previous season, cm), body weight (BW, kg) and social environment (high-low intra-sexual competition; reference level = high competition) and year as random effect. p (> Chi^2^): probability of tests of random-effect terms in the model, each term is removed and REML-likelihood ratio tests computed. *R*^2^_LMM(m)_ marginal variance accounted for the fixed effects; *R*^2^_LMM(c)_ conditional variance accounted for random and fixed effects.

Random effects	variance	sd	p (> Chi^2^)		
year	263.5	16.23	0.277		
residual	195.9	14.0			
**Fixed effects**	**estimate**	**se**	**df**	**t value**	**p**
intercept (high competition)	−35.91	97.326	11.0	−0.369	0.719
NDVI	230.87	163.151	16.9	1.415	0.175
previous antler	0.95	0.126	16.8	7.533	<0.001
BW	−0.08	0.243	14.2	−0.342	0.737
social environment	−16.27	7.141	16.9	−2.28	0.035
*R*^2^_LMM(m)_	0.74				
*R*^2^_LMM(c)_	0.88

The results of the models that incorporated faecal levels of testosterone (Model T, Table 6) and cortisol (Model C, Table 7) produced consistent results with those of Model A. In Model T, the low competition environment had a negative effect on antler size (estimate = −158.61, p = 0.003, Table 6) and males with big antlers in the previous season were also likely to have grown big antlers in the actual season (estimate = 1.1, p = 0.001). The estimates of the effect of testosterone level previous and post exposure to the new social environment were negative and significant (pre-testosterone = −0.45, se = 0.161, p = 0.029; post-testosterone = −1.01, se = 0.232, p = 0.004). Post-testosterone had a significant interaction with social environment, as the levels of post-testosterone increased, antler size decreased in the high competition environment but had no effect under the low competition environment (estimate = 1.05, p = 0.006, Table 6). In this model the random effect year explained no variance and that is why *R*^2^ marginal and conditional had identical values (*R*^2^ = 0.97). There was a non-significant trend for antler size to be associated with high values of NDVI (estimate = 306, p = 0.079) and body weight had no effect on antler size (p = 0.563).

**Table 6:** Coefficients of a linear mixed model (Model T) on red deer antler size against NDVI, previous antler (size of the antler grown in the previous season, cm), body weight (BW, kg), levels of faecal testosterone before exposure to new social environment (pre-testosterone, ng∙g^−1^) and after exposure to new social environment (post-testosterone, ng∙g^−1^) and social environment (high-low intra-sexual competition; reference level = high competition). Other details as in Table 5.

Random effects	variance	sd	p (> Chi^2^)		
year	0	0	1		
residual	66.76	8.171			
**Fixed effects**	**estimate**	**se**	**df**	**t value**	**p**
intercept (high competition)	43.38	53.207	6	0.81	0.446
NDVI	305.99	145.041	6	2.11	0.079
previous antler	1.09	0.113	6	9.64	<0.001
BW	−0.11	0.192	6	−0.61	0.563
pre-testosterone	−0.45	0.161	6	−2.85	0.029
post-testosterone	−1.01	0.232	6	−4.37	0.004
social environment	−158.61	35.046	6	−4.52	0.003
post-testosterone × social environment	1.05	0.255	6	4.11	0.006
*R*^2^_LMM(m)_	0.97				
*R*^2^_LMM(c)_	0.97

**Table 7:** Coefficients of a linear mixed model (Model C) on red deer antler size against NDVI, previous antler (size of the antler grown in the previous season, cm), body weight (BW, kg), levels of faecal cortisol before exposure to new social environment (pre-cortisol, ng∙g^−1^) and after exposure to new social environment (post-cortisol, ng∙g^−1^) and social environment (high-low intra-sexual competition; reference level = high competition). Other details as in Table 5.

Random effects	variance	sd	p (> Chi^2^)		
year	1148.57	33.891	0.011		
residual	30.59	5.531			
**Fixed effects**	**estimate**	**se**	**df**	**t value**	**p**
intercept (high competition)	64.78	60.451	5.9	1.07	0.325
NDVI	426.95	131.364	5	3.25	0.022
previous antler	0.80	0.101	5.0	7.92	<0.001
BW	−0.41	0.150	5.1	−2.77	0.038
pre-cortisol	−0.00	0.005	5.0	−0.92	0.395
post-cortisol	−0.42	0.063	5.0	−6.60	0.001
social environment	−92.13	10.630	5.0	−8.66	0.000
post-cortisol × social environment	0.49	0.080	5.0	6.16	0.001
*R*^2^_LMM(m)_	0.51				
*R*^2^_LMM(c)_	0.98				

In Model C there was an interaction between social environment and antler size, as faecal levels of cortisol increased, antler size decreased in the high competition environment but there was a trend of increase in the low competition environment (interaction post-cortisol × social environment [low competition], estimate = 0.49, p = 0.001, Table 7). In this model antler growth was positively associated with NDVI (estimate = 427.95, p = 0.022), with previous antler (estimate = 0.8, p < 0.001) and negatively related to body weight (estimate = −0.41, p = 0.038). Year explained a significant amount of the variance (p = 0.011) and the model accounted for a large amount of the variance of the system (*R*^2^_LMM(c)_ = 0.98, *R*^2^_LMM(m)_ = 0.51).

The faecal levels of cortisol decreased during the experiment (estimate = −415.5, se = 176.9, p = 0.026) but they were not affected by the social environment, as indicated by the non-significant interaction pre-post × social environment (p = 0.656, Table 8). A simplified model in which the non-significant interaction was removed produced predictions of 476 ng∙g^−1^ and 128 ng∙g^−1^ of faecal cortisol before and after exposure to the new social environment, respectively.

**Table 8:** Coefficients of a linear mixed model on faecal cortisol (ng/g) against time when cortisol was measured (pre- and post exposure to new social environment) and social environment (high-low intra-sexual competition; reference level = high competition and post exposure) and year as random effect. Other details as in Table 5.

Random effects	variance	sd	p (> Chi^2^)		
id	0	0	1		
year	104550	323.3	0.002		
residual	106122	325.8			
**Fixed effects**	**estimate**	**se**	**df**	**t value**	**p**
intercept	557.3	265.15	1.5	2.10	0.201
pre-post	−415.5	176.90	28.0	−2.34	0.026
social environment	77.5	172.13	28.0	0.45	0.655
pre-post × social environment	−101.5	231.31	28.0	−0.43	0.664
*R*^*2*^_LMM(m)_	0.21				
*R*^*2*^_LMM(c)_	0.60				

## Discussion

The results clearly indicate that red deer males that experienced social environments with high intra-sexual competition produced larger antlers and wore down their teeth faster than males living in low male-male competition social environments. By contrast, female rates of tooth wear were similar between HC and LC populations, as they were not driven by same type of intra-sexual competition but likely by environmental conditions that were similar across populations. Furthermore, the experimental design demonstrated that the presence of male rivals induced the growth of bigger antlers as compared to the antlers growth of males in the company of only females. Consequently, our results support the hypothesis that a social environment with a high level of male intra-sexual competition promotes the increase of sex-trait expression with detrimental effect to male longevity.

Our study takes advantage of contrasting management practises that create LC and HC population environments. Previous studies, using genetic and behavioural tools, have shown clear differences in the degree of male-male competition^30^ and the consequences in the transmission of genetic variability to the next generation^29^ between these two social environments. Our results are also in agreement with the effects of male competition in these same Iberian red deer populations on the dark ventral patch, that may be modulated in the short term by urination, changing its size (Carranza *et al*., under review) and its chemical constituents^31^.

Managers may provide supplementary food to these red deer populations, which might introduce confounding effects if supplementation was associated with one of the social environments. However, feed supplementation is occasional, only provided in summer during drought periods and usually in both types of populations^32,33^. In any case, if environmental conditions differed between populations in our study, either due to natural food resources or management practises, we would expect body condition to respond in LC and HC populations in a similar fashion in both males and females, which was not the case. Males of dimorphic species (^9^ and references therein), including red deer^34^, may be more sensitive than females to changes in environmental conditions, but even so, some effect would also apply to females. By contrast, our results show that body weight and tooth wear were similar in females across both types of populations. Additional support to our interpretation comes from the finding that spatial variation in antler length was higher than temporal variation, despite ample evidence that antler size in red deer shows strong inter-annual variation that is weather and density dependent^35–39^. Moreover, if favourable environmental conditions were the main cause for antlers being larger in HC populations, it would be unlikely that males of these populations would show higher rates of tooth wear, as tooth wear is related to harsh habitat conditions in several species of deer^21,40–42^.

Most evidence on factors influencing trait expression comes from variation in ambient environment affecting individual condition^1,3,43^. However, it is increasingly supported that the social environment also influences trait expression not only through regulation of hormone levels^44–46^ but by alternative, little known mechanisms, that might decouple the relationship between hormone level and trait expression^47,47^. Social environment, by means of intraspecific competition, may affect individual condition and hence trait development^8,10^. However, evidence of the effect of social environment, as perceived by individuals, on trait development is scarce. Some examples are related to dominance behaviour, in particular the reduction of trait expression or signalling by subordinate individuals^20,48^. McGraw *et al*.^18^ demonstrated that aggressive interactions experienced during development led to increased size of male sparrows’ badges (*Passer domesticus*). Vergara & Martínez-Padilla^47^ presented an experiment inducing a change in the social environment perceived by red grouse *Lagopus lagopus scoticus* males. They showed that when testosterone-implanted birds increased their comb size, untreated males living in the treatment area decreased their comb size. The authors hypothesised that untreated males may perceive an increase in the competitive ability of competitors and then act themselves as subordinate. Plasticity in costly traits can allow individuals to reduce investment when traits are less useful. An example for a carotenoid-based sexual signal is the decrease in bill colour investment in male zebra finches (*Taeniopygia guttata*) when they are kept in an male-only environment as compared to males living with females, since bill colour is a sexual signal directed to females who use it as a trait in mate choice^16^.

The differences found between our two types of red deer populations may indicate spatial heterogeneity in a landscape of selection intensity^5,30^. It may be suggested that after sufficient generations of divergent conditions one would expect micro-evolutionary changes^49^. Alternatively, reaction norms may allow individual males to modulate the phenotypic expression of a single genotype across a range of environments^3,4,19,50,51^. Continuous hunting exploitation has been reported to produce changes in sex-traits due to the harvesting of animals with the largest trophies^52–55^, sometimes with micro-evolutionary consequences^56,57^. The case presented here could be the first one to show that hunting exploitation leads to a reduction in sex-traits due not to the removal of large trophies themselves but to changes in the population conditions (i.e. structure) that promote males to reduce investment in costly traits. Further research is needed to investigate potential implications of our results, for instance, on reaction-norm thresholds for sex trait development and static allometries^58,59^, as well as potential micro-evolutionary processes^49,57^ affecting sex-trait expression and lifespan under contrasting population structures.

Our experimental design on captive deer produced, for two consecutive years, consistent results with the results found in our wild populations, namely, males produced smaller antlers when they lived exclusively with females, but larger antlers in the presence of male rivals. These results also held when our analyses controlled for faecal levels of testosterone and cortisol metabolites.

The relationship between testosterone and some sex traits has proven to be complex and dependent on the social environment (see above), and similarly occurs in the case of antlers. For example, testosterone is expected to be at its lowest level in the annual cycle, when antlers are cast and during antler growth, however it has a positive role in antler ossification^25^, and a negative relationship has been found between post-rut testosterone levels and antler size^60^. We also found a negative relationship between antler size and faecal testosterone in samples taken pre- and post the period of antler growth. For post-testosterone, there also was a significant interaction showing that the negative relationship with antler size occurred only in conditions of high male-male competition. Males with larger antlers in HC environment showed low testosterone levels in agreement with the expectation, while variation in antler size and testosterone were higher among LC males. Results for the dark ventral patch in Iberian red deer populations showed a positive relationship between this trait and testosterone, but only in HC populations (de la Peña *et al*.^61^) Both results suggest that the relationship between testosterone and sex traits may be weaker when sexual competition is lower, but further research is needed.

We used faecal cortisol as a proxy of chronic stress caused by the social environment, under the prediction that stress could be the cause of smaller antler production. Clearly, the results showed that differences in cortisol between treatment populations were not associated with antler size.

All in all, our results strongly indicate that the contrasting conditions in male age-structure and adult sex-ratio between both types of populations are the most likely cause for the differences found in sex-trait expression in males, and its concomitant effect in tooth wear. These results have important implications not only for the evolutionary theme of sex-trait expression related to the social environment, but also as a driver of sexually dimorphic senescence.

LC and HC populations differed in the rate of tooth wear; tooth depletion has been reported as one main cause of senescence in ungulates^23,25–28,62,63^. We assumed that differences in tooth wear between populations and sexes were not due to differences in enamel and dentine hardness^64^. It is unlikely that across 56 populations differences in tooth hardness were associated to the management types that produced our two male-male competition environments. Furthermore, it has been demonstrated in Scottish red deer that male and female molars do not differ in enamel and dentine hardness^64^. Our results suggest differences between populations in sexually dimorphic senescence. We were unable to obtain a large dataset of old males from LC populations, because few males survived beyond middle age due to hunting. However, the differences in the rate of tooth wear indicate that LC males not only invested less in antlers but also experienced delayed tooth depletion compared to HC males.

Sexual selection has increasingly been acknowledged as the main force responsible for sex differences in senescence and longevity in different taxa including ungulates^28,65–68^, so the level of intra-sexual competition is expected to affect male senescence. Our data show that males in populations with a female-biased sex ratio, where they can mate from early age but with little intra-sexual competition^29,30^, experienced delayed senescence, as measured by tooth wear. This is in line with the findings that red deer males that were involved in competition early in life experienced early senescence^69^. For moose, *Alces alces*, it has been shown that the mean weight of males living in a strongly female biased population decreased more precociously than that of males living in population with a more even sex ratio, which was interpreted as early senescence^70^. We also found early peaking and decrease of body weight in our female biased red deer populations, although our results on tooth wear do not suggest that these differences in body weight were related to senescence. Rather, body weight, as well as antler size, are needed for male-male competition and both have consequences in tooth wear and senescence. Therefore, earlier reduction of body weight in a female-biased environment may denote a different strategy rather than a form of male cost potentially related to exhaustion, since it is associated with lower tooth wear, while males in a high-competition environment have to invest in body weight and antler size at the expense of lifespan.

Classic theory predicts that the sex experiencing a higher rate of extrinsic mortality evolves faster aging and reduces its longevity^71^. Regardless of an insufficient number of generations under current conditions, our results may seem paradoxical with this prediction, since males of LC populations experience very high near-random age-specific mortality rates due to hunting, thus resembling a case of strong extrinsic mortality for males below their prime age^33^. The maintenance of such conditions in exploited animal populations has led to advanced age at first reproduction and earlier senescence^49,72–74^. Perhaps we should expect earlier senescence in LC populations if there were no differences in male mating strategies and investment in costly sex-traits in both types of populations. However, the lower investment in antlers by LC males with the consequences of decelerated tooth depletion might act in the opposite direction to the former prediction of an earlier senescence due to extrinsic mortality. We may speculate about different processes taking place in these populations. On one hand, early reproduction and higher extrinsic mortality of males in LC populations would favour early senescence under a micro-evolutionary process according to theory^71^. On the other hand, breeding under conditions of low intra-sexual competition would favour smaller sex-traits with lesser costs involved, thus delaying senescence, in this case under a process of behavioural and physiological plasticity on the basis of reaction norms. The prevalence of the effect in one or the other direction may depend on the micro-evolutionary stage. Hunting areas in Spain have remained under similar conditions to those studied here only over the last 30–40 years. In addition, although fences do restrict deer movement between estates, it is unlikely they are completely gene flow impervious, as suggested by the results of Martínez *et al*.^75^ and Pérez-González *et al*.^76^. Deer are keen to walk along fences and find holes to slide through, which may prevent genetic isolation and local adaptations^77^. Thus, we believe that the potential for higher longevity favoured by reduced tooth wear in LC males should likely be the consequence of individual plasticity without any significant role of micro-evolutionary change.

## Methods

To test our hypotheses we applied two approaches, (1) a natural experiment comprising measurements of animals legally culled in 56 red deer populations, which can be neatly classified into two levels of male-male competition for mating opportunities, and (2) an experimental design in which captive males were exposed to two social environments, (i) male living exclusively with females, (ii) male living with other males (no females), during the period of antler formation.

### Natural experiment

We used information from 6696 (2495 females and 4201 males) Iberian red deer culled on 56 hunting estates (populations) in the centre-west and centre-south of Spain between 1997 and 2009. The size of individual hunting estates ranged between 750 and 3000 Ha, the habitat included mountain ranges covered by Mediterranean shrub (*Cistus* spp., *Erica* spp., *Arbutus unedo*, *Phyllirea* spp., *Genista hirsuta*, *Lavandula* spp.) and trees (*Quercus* spp., *Olea europaea*), along with lower, flatter land, and open oak woodland known as ‘dehesa’^76^. All deer used in this study came from legal game activities, shot under the modality of big game driven hunt, known as Spanish ‘montería’^39,75^. This type of hunt has been shown to be a good procedure to obtain unbiased samples of deer populations^78^. These estates were under two contrasting types of management: stock-proof wire mesh fenced estates, versus unfenced grounds in which deer could migrate^29,33^. Fenced hunting estates focus deer harvesting on adult males. In contrast, in unfenced hunting estates few males are allowed to reach old age, as no hunter risks sparing from culling sub-adult males as they could be shot in adjacent estates if they move out^33^. In unfenced estates most sub-adults are yearlings, as it is illegal to shoot them^33^. As a consequence, the sex-ratio and age structure within unfenced populations are strongly biased towards females and young males, compared to fenced estate populations^29,33^. Therefore, on fenced estates males experienced higher level of intra-sexual competition compared to that on unfenced estates, where virtually all males can mate even if they are sub-adult^29,30^. We refer to fenced and unfenced estates as populations with high (HC) and low (LC) levels of competition for mating opportunities.

The information used was body weight (BW, intact animal with gut minus blood from bleeding, ± 0.1 kg), fresh right antler beam length (AL, ± 1 cm); mandible length (ML, ± 0.1 cm); thickness of the dentine of first lower molar M_1_ (molar height, MH, ± 0.1 mm) as a proxy of dental wear;^64^ sex; age (in years, see below); shooting date and location at estate level. The preparation of the jawbone and sectioning of M_1_ was carried out following Pérez-Barbería *et al*.^24,25,79^ MH was estimated by measuring on a coronal (frontal) section of M_1_, with the aid of a calliper and a magnifying glass, the thickness of the dentine from the top of the cementum of the radicular pad to the middle point of occlusal surface^23,24,64^. Tooth wear was assessed as the negative relationship of MH with age^24^; the advantages of recording MH as dentine height against other measurements of molar height as a proxy of tooth wear is described in Pérez-Barbería *et al*.^25,64,79^. Mandible length was measured in the laboratory from the mesial border of the first incisor socket to the vertical part of the ramus, after having removed the flesh at these two points. We used mandible length as a measurement to control for animal size, independent of body condition. Besides, controlling for mandible length also makes it possible for MH to be a reliable proxy of tooth wear as mandible length covariate controls for potential differences in molar size between animals of different size^24,25,64^. The length of the main beam of the right antler was measured on the animal carcass within a few hours after being shot, from the centre of the lowest outside edge of the burr over the outer side to the most distant point of the main beam. The point of the burr was where the centre line along the outer side of the beam intersected the burr. Measurements of broken antler beams were discarded.

The age in years was estimated by counting the milky-coloured cement layers on the root pad (assuming 1 layer per year of age) of the coronal section of M_1_, aided by a reflected light microscope^80,81^. When M_1_ was missing, or the cement layers were poorly defined, M_2_ was used and the age in years was estimated as the number of cement layers plus one. This age estimation technique provides an acceptable approximation to the actual age for population studies^82^.

We collected information of the habitat quality of each estate following the index described in Pérez-González & Carranza^29^; this information was collected during 2004 and 2005, and we assumed that the relative differences in the index, between fenced and unfenced estates, remained similar across years. This is because this index is mainly based on changes in the mosaic composition of Mediterranean shrub vegetation, which is likely to have changed little during the sampling period of this study. To assess for differences in age structure of fenced and unfenced populations that could account for differences in sexual competition and level of polygyny between both types of populations, we used the age structure of shot deer, and the proportion of males older than 2 years of age from direct counts carried out during 2004 and 2005 rut, as described in^29,30,33^. The latter was used to corroborate that the age structure of shot deer was not affected by differences in hunting selection between HC and LC populations, as their relative differences held when using direct counts data.

Artificial selection by biased hunting could be a potential cause of differences in antler size between populations. To assess if hunting targeting large antlers could be the cause of smaller antler size in LC estates vs. HC estates, we used the following reasoning and approach. Most males culled in LC estates were 2 years old (see above); if hunters in LC estates aimed to shoot 2 year-old males with large antlers, then, 2 year-old individuals that were not culled should be those with smaller antlers. If this is true, an interaction between age (only 2 and 3 year old deer) and population type (LC, HC) would be expected, with LC 3 year old males having smaller antlers than those of the same age culled in HC estates. To assess this hypothesis, we fitted a mixed linear model with antler length as the response variable and the interaction between type of estate (LC, HC) and age as the fixed effects (only males of 2 and 3 years of age), and as random effects population and hunting season (see below for details on the statistical analyses).

### Captive deer experiment

#### Study area

The experiment was carried out at Lagunes Game Farm (Southern Spain) during 2017 and 2018, using 234 individuals distributed into 7 experimental plots. The experiment was repeated across two consecutive years in order to increase sample size to overcome constraints on the number of animals and plots. We used twenty-four 4 year-old red deer individually marked with ear-tags to enable identification. Eighteen males constituted the high competition (HC) group (control), which was allocated to one of the plots. Six low competition (LC) groups were constituted by one male and between 13 and 21 females in each group allocated randomly to a different plot. The HC group of males resembled a situation with “few” females since there were females in other enclosures separated less than 0.5 km in the farm. Males could not see the females but could likely sense their presence via scent signals from the distance. We avoided keeping females with many rutting males in an enclosure since it would have been unsafe for the females, leading to ethical concerns. This design was appropriate for our purpose of creating a clear difference between HC and LC social environments. A similar experimental arrangement was repeated the second year using a different batch of experimental animals. Groups were moved into the plots in March, just after antlers were cast, and the experiment finished in July when antlers were fully grown and velvet was shed.

#### Antlers measurements

Because it was not possible to weigh the antlers of live stags, we used a measure of antler size of fully-grown antlers. This measure was computed as the summation of all lengths and perimeters of right and left antlers that are recorded to assess the quality of red deer trophies. These were: perimeter of the pedicle, perimeter of beam between the brow tine and bez tine, perimeter of the beam between bez tine and crown, beam length, brow tine length and bez tine length.

#### Primary productivity

In order to have an objective measure of the quality of the vegetation in each plot through the experiment, primary productivity was assessed using the Normalized Vegetation Difference index (NDVI), which has been widely used as a proxy of quality and quantity food resources in ungulates^83–85^. NDVI was calculated using QGIS^86^ and images from Landsat8 provided by Copernicus Global Land Service with a 30×30 spatial resolution, corrected by atmospheric perturbation using Dark Object Subtraction in the semi-automatic classification plugging^87^. Finally, as a proxy of quality and availability of browse and grazing plants within plot and experimental year, all NDVI values available and with less than 20% cloudiness were averaged from April to July (2017: 07 April, 24 April, 09 May, 10 June, 26 June, 12 July, 28 July; 2018: 26 April, 13 June, 29 June, 31 July).

#### Faecal steroid hormones

Faecal grab sampling was conducted on individual deer from the rectus at the beginning and end of the experiment (March and July). Faecal samples were processed to quantify the metabolite levels of testosterone and cortisol (Iberian red deer^88^; roe deer *Capreolus capreolus*^89–91^. Frozen faecal samples were dried and milled. A solution of 2 ml of phosphate buffer and 2 ml of 80% methanol were added to 0.5 g of faecal material and the mixture was vortexed. Samples were on a shaker for 16 h, the solvent was decanted and the supernatant was centrifuged at 4000 rpm for 30 min. Faecal metabolite hormone levels were determined using a commercial enzyme immunoassay kit (testosterone: DEMEDITEC DEH3388; cortisol: DEMEDITEC DE1559). For simplicity we refer to testosterone and cortisol throughout the text meaning faecal metabolites of these hormones.The testosterone intra-assay coefficient of variation was 10.8% and inter-assay 10.6%. The cortisol intra-assay coefficient of variation was 9.2% and inter-assay 10.2%. Standard dose-response curves were constructed by plotting the binding percentage against the standard hormone concentrations. The results were expressed as nanograms per gram of faecal dry matter.

#### Ethical statement

All our methods were performed in accordance with relevant guidelines and regulations. The experimental protocol was approved in agreement between the Wildlife Research Unit at the University of Cordoba and Lagunes Farm. Lagunes Game Farm complied with the Spanish animal welfare legislation, it was daily attended by qualified personnel and a veterinary looked after the animals regularly. Ear tagging, body weight monitoring and faecal grab sampling were procedures that did not require animal experimentation license as they were carried out as standard farming activities to assess animal health and condition. Data obtained for this study derived from normal farming activities.

### Statistical analysis

#### Natural experiment

We used linear mixed-effects regression models to predict the responses of beam antler length, body weight, molar height and habitat quality, against a number of predictors, and their meaningful interactions, in LC and HC populations, and controlling for population and hunting season (October to February) as random sources of variation. Generalised additive mixed linear models GAM^92^ were used in an explorative stage of analysis to corroborate that regression smoothers with basis of modest size were appropriated to model the data. Consequently, age and mandible length were fitted as orthogonal quadratic terms in mixed linear models because of their simplicity when testing for interactions in comparison with GAM functions with basis of modest size. Besides, the quadratic function is a convenient representation of many life history traits related to body condition that increase with age until they reach a peak and decrease in the last stages of life^25,93^. As body weight is a condition-dependent variable affected by the time of year when the animal was shot, we fitted two complementary circular functions as covariates to account for changes in body weight across the hunting season, following the approach used in Pérez-Barbería *et al*.^24^. The circular functions were sine DOY and cosine DOY, where DOY was the day of the year in which the animal was shot. For practical reasons (i.e. starting of the hunting season) we arbitrary fixed 26^th^ of September at DOY = 1, changing the starting reference point does not affect the results of the analysis.

The age structure of males and females in HC and LC populations were plotted as kernel densities (i.e. similar to a continuous histogram), using the Gaussian function as smoothing kernel. On these plots we superimposed the predicted mean age for HC and LC populations calculated by separate mixed linear models for each sex, with populations as fixed effects and hunting season as random effect.

Normality and homoscedasticity were verified. We used bivariate plots of the terms in the models and plots of fitted values against residuals in order to detect mistakes in our records. Some odd values could be identified as typing mistakes and were corrected in the database, and fourteen odd records were removed from the dataset^94^. The full models were simplified using backward elimination by removing the non-significant fixed-effects terms, one at a time, following the principle of marginality (i.e. the highest order interactions were tested first and if they were significant, then the lower order effects were not tested for significance). Backward elimination was based on p-values in favour of information theory approaches, like ΔAIC or BIC^69^, as successfully used in Pérez-Barbería *et al*.^24,25^. The coefficients of the final models were calculated using Restricted Maximum Likelihood REML^70^, and the degrees of freedom in the estimate of the coefficients of the mixed linear models followed Satterthwaite’s approximation^95^, since in linear mixed-effects models, determining the “correct” value of degrees of freedom in the estimate of the coefficients is meaningless^92,94^. The variance explained by the models was represented as *R*^2^ marginal (variance accounted for by the fixed effects; *R*^2^_LMM(m)_) and *R*^2^ conditional (variance accounted for by random and fixed effects; *R*^2^_LMM(c)_), following a method developed for linear mixed-effects models ^94^ and used in Pérez-Barbería *et al*.^24,25^. All analyses and graphics were conducted in R software^96^, mainly using lme4^97^, lmerTest^95^ and mgcv^98^ packages.

#### Captive deer experiment

We modelled the response of antler growth to social environment (LC vs. HC) using three independent linear mixed regression models. The first model (Model A) fitted as fixed effects social environment, body weight (BW), NDVI and antler size of the antler grown in the previous season (previous antler), and year as a random effect. Previous antler was included in the model as it was expected that males potential to produce big antlers remained across their life independent on the environmental conditions. BW was fitted in the model to account for a positive relationship between animal size and antler size, although we expected this relationship to be weak as all animals were of the same age and the differences in body weight were small as they were drawn from the main herd following a criterion of similar body size. A second model (Model T) was the same as Model A, adding two new terms, the faecal levels of testosterone when the animals were deployed in the plots (pre-testosterone) and at the end of the antler growth period (post-testosterone), together with the interaction post-testosterone × social environment. We kept to a minimum the number of interactions in the model to avoid overparameterization as the sample size was small. A third model (Model C) was fitted as Model T but replacing faecal testosterone with faecal cortisol. Models A, T and C were used to predict antler size after animals were exposed to the new social environment, and the predictions were plotted on the condition of the fixed effects set to their mean values (only shown for Model A). Models were fitted using the R software packages described in Natural experiment above.
